# Distraction of attention by novel sounds in children declines fast

**DOI:** 10.1038/s41598-021-83528-y

**Published:** 2021-03-05

**Authors:** Nicole Wetzel, Andreas Widmann, Florian Scharf

**Affiliations:** 1 Leibniz Institute for Neurobiology, Brenneckestr. 6, 39118 Magdeburg, Germany; 2 Center for Behavioral Brain Sciences, Magdeburg, Germany; 3 University of Applied Sciences Magdeburg-Stendal, Magdeburg, Germany; 4 Leipzig University, Leipzig, Germany; 5 University of Münster, Münster, Germany

**Keywords:** Psychology, Human behaviour

## Abstract

New task-irrelevant sounds can distract attention. This study specifies the impact of stimulus novelty and of learning on attention control in three groups of children aged 6–7, 8, and 9–10 years and an adult control group. Participants (N = 179) were instructed to ignore a sound sequence including standard sounds and novel or repeated distractor sounds, while performing a visual categorization task. Distractor sounds impaired performance in children more than in adult controls, demonstrating the long-term development of attention control. Children, but not adults, were more distracted by novel than by repeated sounds, indicating increased sensitivity to novel information. Children, in particular younger children, were highly distracted during the first presentations of novel sounds compared to adults, while no age differences were observed for the last presentations. Results highlight the age-related impact of auditory novel information on attention and characterize the rapid development of attention control mechanisms as a function of age and exposure to irrelevant novel sounds.

## Introduction

Attention plays an essential role in many cognitive functions, as it controls the flow of information between individuals and their environment. The ability to selectively attend determines successful learning and influences academic performance^1^. When focusing on a task, attention can be distracted by new, unexpected, but task-irrelevant events (e.g., smartphone sound; note, here, *new* events refer to uniquely occurring environmental sounds as presented in the experiment). Novel events can capture attention, require memory resources, and have an impact on motivation and behavior. Novel events are significant, as they might announce important changes in the environment that could require an immediate response. The processing of unexpected novel sounds evokes a cascade of neural activity in widespread cortical and subcortical networks [for review see^2^]. This can result in an involuntary allocation of attention toward the novel event^3^, prioritized processing of the novel event^4^, and enhanced memory formation^2^.

Distraction of attention by unexpected and task-irrelevant sounds can be described by a three-stage model of involuntary attention [e.g.,^5,6^]. The first stage of the model includes a prediction of the acoustic environment that can be violated by unexpected sounds^7^. In the second stage, this violation can trigger an involuntary orienting of attention toward the new sound, followed by voluntary reorienting to the primary task, given an adaption of behavior is not required. It is assumed that when attention is oriented toward new, but task-irrelevant events, less resources are available to perform the task at hand resulting in impaired performance, that is, prolonged reaction times or decreased hit rates [distraction effect, for review see^8^]. This model is well established in auditory attention research with adults but less investigated from a developmental perspective [for review see^9^], despite the fact that the detection and evaluation of novel events occurring outside the focus of attention is particularly important for children, because novel events can enhance knowledge about the world. In line with the long-term developmental trajectory of brain regions involved in attention control such as the prefrontal cortex^10,11^, several studies on behavioral and brain level indicate that the control of attention in the presence of novel sounds develops throughout childhood [for review see^9^].

The ability to focus attention and ignore task-irrelevant novel sounds develops considerably from age 4 to 6 years^12^. In middle childhood, usually during elementary school years, developmental studies reported inconsistent results on attention control. A number of studies presenting a sequence of frequently presented and repeated standard sounds and infrequently presented unexpected novel or deviant sounds reported increased distraction effects in younger than older children in response to novel or deviant sounds in the middle childhood [e.g.,^13–15^]. Immature attention control in children aged 7–10-years was also reported by studies using different approaches, e.g. a Duplex-mechanisms approach [^16^, but see^17^]. However, some studies did not report decreased distraction effects with increasing age in response to novel sounds in the middle childhood^18–21^. The discrepant findings could result from different examined ages groups and by differences in experimental conditions such as modality (auditory-visual, auditory-alone), by differences in the information provided by distractor sounds, or the number of presented distractor sounds. In a recent study, unexpected and task-irrelevant sounds, that where either new environmental sounds or repeated pitch deviant sounds, were rarely and randomly presented within a sequence of repeated standard sounds. At the same time, children aged 7–10 years performed a visual categorization task^15^. Distraction effects declined with increasing age and performance was more impaired by novel than by repeated deviant sounds. In addition, a decline of distraction effects in response to novel but not to pitch deviant sounds during the experimental session in the youngest children was reported. The authors concluded that separate mechanisms underlie the processing of different types of distractor sounds that follow differing developmental timelines^15^. We hypothesize that the novelty of task-irrelevant sounds could be a relevant and age-sensitive factor influencing children’s distraction of attention. We assume that children are initially much more distracted by task-irrelevant sounds than adults, but that distraction effects decrease during the experimental session. In addition to age, novelty is expected to be an important factor that modulates the decline of distraction effects during exposure. In sum, we assume that children are initially more distracted by task-irrelevant sounds than adults and that this effect is particularly pronounced for novel sounds.

**Figure 1:**
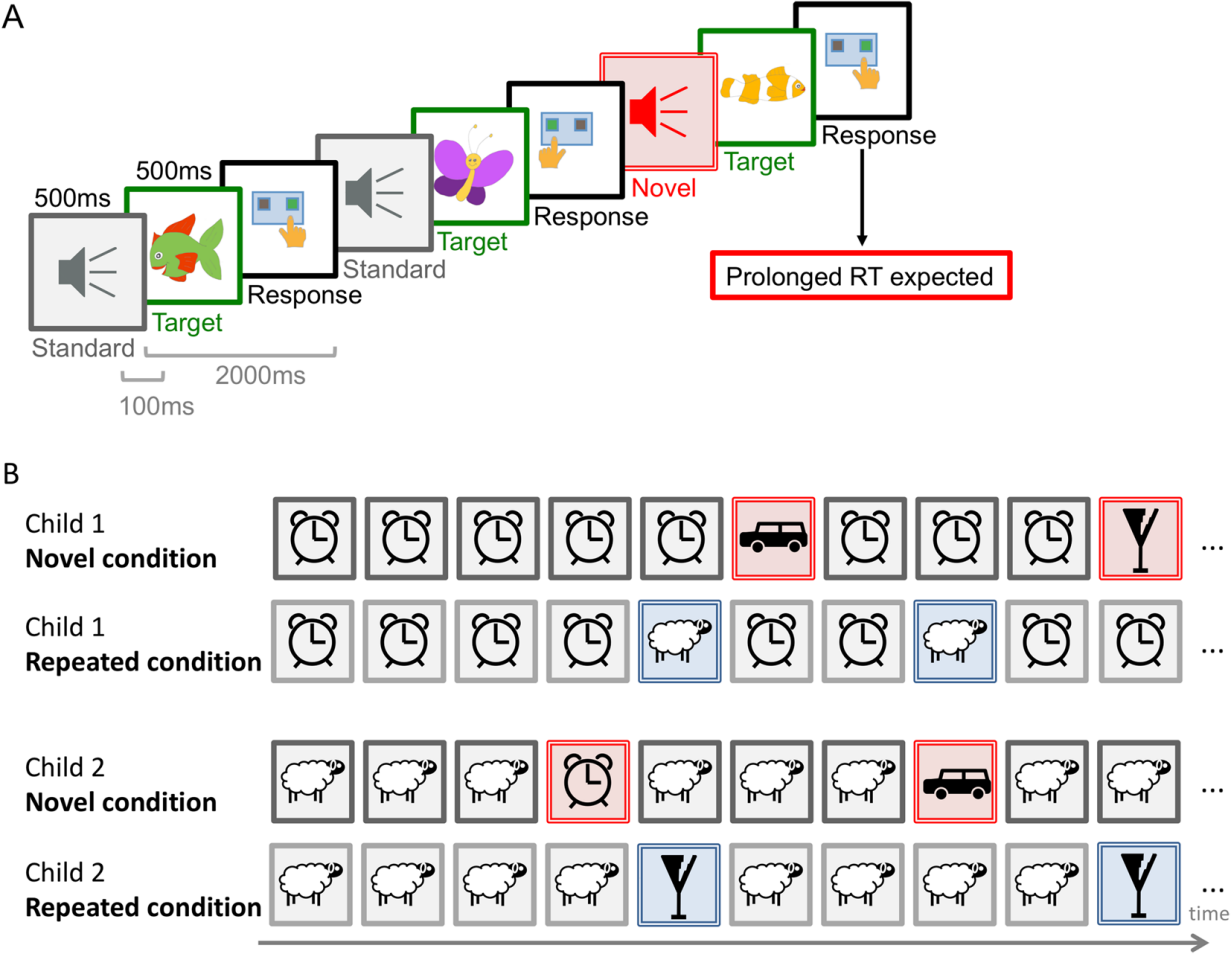
Distraction Paradigm*.* (**A**) Participants distinguished target categories by button press. In the displayed version they were asked to press the right button when a fish appears and the left button when a butterfly appears. Each target (green) was preceded by task-irrelevant sounds that could be either frequently presented standard sounds (grey) or rarely presented distractor sounds (new (red) or repeated (blue)). Distractor sounds delayed responses to the upcoming target. (**B**) Examples of sound sequences in the novel condition (different distractor sounds (red) were presented uniquely) and in the repeated condition (the same distractor sound (blue) was presented repeatedly). The standard sound varied between participants.

To determine the impact of novelty on attentional distraction from a developmental perspective, we compared a condition in which several environmental distractor sounds (novels) were presented only once with a condition in which environmental distractor sounds were presented repeatedly in a sequence of repeated standard sounds (Fig. 1). Three groups of children aged 6–7, 8, and 9–10-years and an adult control group performed a visual categorization task. We measured reaction times (RT) and response accuracy. We expected prolonged RTs in the categorization task when a novel or repeated distractor sound preceded a target, demonstrating distraction of attention. Second, we hypothesized that distraction effects decrease with age indicating development of attention control. Third, RTs were expected to be longer in response to novel sounds, than to repeated sounds. This effect of longer RTs following novel sounds was expected to be pronounced at the beginning of the experiment in younger children. In terms of everyday life, results could imply that unexpected new sounds in the classroom distract attention much stronger in young children than estimated by previous studies, that averaged distraction effects across the experimental session. Results could also explain inconsistent findings on age effects of attention control in middle childhood.

## Method

### Participants

A total of 196 participants participated in the study (see Supplement Part C, for detailed sample size considerations). Most of the children were tested in after-school care centers in a mid-size city. The remaining participants were tested at the institute. To investigate distraction effects throughout childhood, children were divided into three age groups: 6–7 years, 8 years, 9–10 years. Furthermore, an adult group was investigated to compare children and adults. A total of 17 participants were excluded from the analysis, because of the following reasons: 5 children were picked up from the after-school care center by their parents during the experiment, who did not want to wait for their children to finish the experiment (one 6-year-old, two 8-year-olds, two 9-year-olds), 4 children did not perform the complete number of blocks, 6 children (one 7-year-old, two 8-year-olds, three 9–10-year-olds) and 2 adults reached a hit rate lower than 2 standard deviations from the mean of the respective age group. Data of 54, 6–7-year-olds (mean age 7 years, 3.5 months, 32 female), 37, 8-year-olds (mean age 8 years, 4.4 months, 17 female), 50, 9–10-year-olds (mean age 9 years, 8.0 months, 29 female), and 38 adults (mean age 24 years, 2.4 months, 32 female) were analyzed. The sample included only German-speaking participants. Children gave informed oral consent. Parents and adult participants gave written informed consent. Participants and parents denied any medication affecting the central nervous system, neurological disorders, hearing disorders, or attention deficit hyperactivity disorder. Participation in the study was rewarded by vouchers for a local toy shop (children) and by money or course credit points (adults). All experimental protocols were approved by the ethics committee of the Medical Faculty of the Leipzig University and the study was conducted in accordance with the Declaration of Helsinki.

### Stimuli, task, paradigm

Participants were asked to distinguish two different visual target categories by fast and correct button presses. Each target was preceded by a sound that was irrelevant for the discrimination task and was to be ignored. In two conditions, the novelty of the task-irrelevant sounds was varied (novel condition, repeated condition).

#### Sounds

Sounds were identifiable environmental sounds, for example sneezing, bird song, fragment of a gong, etc. [database described by^22^]. Sounds had a duration of 500 ms including a raised cosine windowed fade-in and fade-out of 10 ms each. Sounds were presented with an average loudness of 53 dB(A), 55 dB SPL. Loudness of sounds was equalized to 6.5 sone [DIN 45,631/ISO 532B with diffuse field equalization^23^].

#### Targets

The novel and the repeated condition consisted of three blocks each. Per block two different visual target objects were presented pairwise: (1) princesses and knights or (2) cats and hens or (3) butterflies and fish. Each visual target category consisted of two variants, one oriented slightly toward the left and the other toward the right side. For example, a cat was either brown and facing left or black and facing right. Each version of the target objects was presented blocked with a probability of 25% in a pseudo-randomized order. To maintain the children's motivation and interest in the task, the target objects were presented with different background landscapes. Princesses and knights were presented in front of a palace and a fortress, cats and hens were presented in front of a basket and a hen-roost in a village, and butterflies and fish were presented together with a flowering shrub and a pond embedded in a coastal landscape.

#### Session

A novel condition (uniquely presented novel sounds embedded in a sequence of standard sounds) and a repeated condition (one repeated distractor sound embedded in a sequence of standard sounds) were presented in a blocked manner. Each condition included three blocks (princesses/knights—cats/hens—butterflies/fish). The visual targets did not differ between blocks.

Every child was introduced to the procedure first and then the experimenter asked whether the child wanted to perform the task and answered any questions. Participants were asked to press one of two buttons allocated to the target categories respectively (Fig. 1) and were asked to respond as fast and correct as possible when a target appears. They were instructed to ignore the sound sequence and focus only on the visual task. The instruction was given in the context of a story in that the children could help the target objects to reach their home as fast as possible. Participants were asked to press the left and right buttons using left and right index fingers. Correct responses within the response window of 2 s after target onset were rewarded by target objects flying or jumping to the preferred place, for example, the fish moved into the pond and the butterfly flew to the flowers. Movement to the preferred place was realized in a stop-motion animation of 600 ms duration starting immediately after a correct response but not earlier than 200 ms after visual target offset. The first button press for every trial was registered.

Each of the three blocks per condition was preceded by a specific training block. The training blocks introduced the respective targets and included six trials of standard sounds (the same standard sound as in the experimental blocks) and two distractor sounds (novel or repeated sounds as in the subsequent experimental block). Novel sounds in the training block (n = 2, novel condition) were not repeated in any experimental block, while repeated distractor sounds were the same in the training and experimental blocks (per participant). There was no time limit for responses in the training block.

The novel and repeated condition were presented blocked in a within-subject design. Condition and block order were counter-balanced across participants. In 77.8% of the trials the standard sound was presented (n = 28 per block, n = 84 per condition). In 22.2% of the trials a distractor sound was presented (n = 8 per block, n = 24 per condition). In the repeated condition the same distractor sound was presented 24 times, while in the novel condition 24 different novel sounds were presented. The standard sound was the same in both conditions.

The effects of physical sound features and the meaning of sounds were controlled for by a pseudo-balanced order in which each sound served as standard, repeated distractor and novel distractor. For example, for participant 1, sound 1 was defined as standard for both conditions and sound 2 was defined as repeated distractor sound in the repeated condition. The remaining sounds 3–26 were presented uniquely (novel condition). For participant 2, sound 2 was defined as standard, sound 3 was defined as repeated distractor, sounds 1, 4–26 were defined as novels and so on. The combination of sounds was the same for the respective index of participants in each age group, that is, the first child in each age group was presented with the same combination of standard, repeated, and novel sound (standard = 1, repeated = 2, novels = 3–26). The order of sound types (standard, distractor) was pseudo-randomized for each participant with the restriction that each distractor was preceded by at least two standard sounds. The allocation of standards and distractors to the two target categories was balanced.

The trial onset asynchrony was 3.3 s. Each trial started with a sound (500 ms), followed by a target (500 ms) 100 ms after sound offset. In case of correct responses within a 2 s response window, the feedback was presented immediately after the response, but not earlier than 200 ms after target offset, for 600 ms. In cases of incorrect or missing responses, no feedback was presented during those time intervals.

Each block consisted of 36 trials and had a duration of about 2 min. The session lasted 20–30 min including breaks. If parents agreed, sweeties were offered in the break time between the blocks. The experimental paradigm was presented using a MacBook (13.3″) with Matlab (The MathWorks, U.S.A.) and Psychophysics toolbox^24^. Responses were collected using two large external response buttons placed on the left and the right side of the MacBook. Response buttons were connected with a RT-Box which provided precise response time measurements^25^.

### Data analysis

The first two trials per block were removed from analysis, because the establishment of a rule requires the presentation of at least two standard sounds^26^. Invalid trials were defined as trials without responses within the response time window (2 s after target onset) and trials including responses faster than 100 ms after target onset. Invalid trials were removed from reaction time and hit rate analyses. Only correct responses within the response time window of 2 s after target onset were subjected to reaction time analyses. The first standard after a distractor was removed, as it is frequently affected by distractor sound processing^27^. The analyses were performed using Matlab (The MathWorks, U.S.A) and the programming language *R*^28^. Mixed effects models were estimated using the packages *nlme* (RT analyses), *lme4*^29^*,* and, *lmerTest* [hitrate analyses^30^]. Linear mixed effect models (LMMs) offered two main advantages over simpler models, such as analyses of variance, for the present purposes: First, LMMs do not require averaging over trials within each participant, enabling us to estimate an individual distraction effect for each participant. Such individual estimates are extremely informative because they allow for detailed analyses of the consistency of experimental effects across participants^31^. Second, LMMs allowed us to account for inhomogeneous variance between groups as adults’ response times may vary less across trials than children’s response times^32^. Additional model checks indicated no further cause for concerns regarding model assumptions such as normality of the residuals.

We are aware that despite the increasing usage of LMMs in recent years, many readers may not be familiar with all details regarding LMM model parameters and their interpretation and that this might make it difficult to interpret our LMM results, especially when there are as many model parameters as is the case here. Therefore, we decided on a two-fold strategy for a comprehensive presentation of results. First, for all models, we present statistics that many readers are familiar with such as ANOVA-like tables with (type-3-) *F*-Tests. Second, we present model-implied (= predicted) values or distraction effects for all factor combinations throughout the results section. That is, we modelled the dependent variable (i.e., RT or correctness of the response) as a function of the full set of predictors, including all possible interactions so that these allow the *estimation* of the hypothesized effects^33^. Then, we extracted the model-implied response times or distraction effects, respectively, for all levels of the predictor variables (e.g., predicted RT for 6–7-year old children to standards in the repeated condition) and calculated 95% confidence intervals to asses estimation accuracy. These model implied values may be interpreted as means of the dependent variable (i.e., RTs or hit rate) or differences of means (distraction effect: distractor minus standard trials), respectively. For the full model output including all raw model parameters, the interested reader is kindly referred to the Supplement. To illustrate the consistency of RT differences between experimental conditions across participants (i.e., variance of the random slopes), we computed individual random effect estimates for the random slopes of the factor *Sound* (i.e., distractor vs. standard) and plotted these against the respective fixed effect estimates (i.e., Fig. 3).

As measures of model fit, we calculated marginal and conditional R^2^, that is, estimates of the variance explained by the fixed effects or by all fixed and random effects together, respectively^34^. Additionally, we report all distraction effects both in the original units and on a standardized scale. For the RT analyses, we standardized the model parameters by standardizing the dependent variable (i.e., RT) across all trials and participants and refitting the model with the standardized RT. This offers a straight-forward interpretation of the standardized distraction effects. For instance, a standardized distraction effect of 0.3 represents a difference of 0.3 *standard deviations* in response times when a distractor versus standard sound was presented. For the hit rate analyses, we report odds ratios (ORs) accordingly.

Two linear mixed models were specified with response times as dependent variable in which participants were treated as clustering variable (“level 2”) and trials were the primary units of investigation (“level 1”). The first model (*condition effect model*) aimed at investigating differences in the response times between the experimental conditions and age groups. Response times were modeled as a function of the categorical predictors *Sound* (Standards vs. Distractors), *Condition* (Novel vs. Repeated), and *Age* (6–7, 8, 9–10 years, adults) as well as their interactions. The model included a random intercept and random slopes for the predictors *Sound* and *Condition* and their interaction. That is, ‘average’ response times for all combinations of *Sound* and *Condition* were allowed to vary across participants. The second model (*block effect model*) aimed at investigating the trajectory of the distraction effects across the experimental blocks. To do so, response times were modeled as a function of *Sound* (Standards vs. Distractors), *Age*, and (experimental) *Block* (1, 2, …, 6). *Block* was treated as a categorical predictor, because no specific trajectory for the development of response times and distraction effects across blocks was especially justified from a theoretical perspective (especially, a linear trajectory across blocks seemed inappropriate, first, because learning curves tend to be non-linear in general, and, second, because the switch of conditions after half of the blocks made it difficult to predict a specific trajectory across the experiment). In addition, *Randomization* (Novel condition first presented vs. repeated condition first presented) was included as a predictor to account for the fact that half of the participants started the experiment with the novel condition whereas the other half started with the repeated condition. A random intercept as well as a random slope for *Sound* were included into the random effect structure. For both models, the residuals on the level of the trials were allowed to be heteroscedastic across age groups to account for potential differences in the trial-level variability across age groups (e.g., more variability of response times in younger children).

Hit rates were also analyzed treating the single trials as primary units of observation. At the level of the single trials, the quality of the response is a dichotomous variable (0 = incorrect response, 1 = correct response). To account for the binary dependent variable, a generalized linear mixed effects model with a binomial distribution of the outcome and a logit link function was estimated (i.e., a “multilevel logistic regression”). With regard to the predictors and also for response times, the same models were estimated. However, only a random intercept representing individual differences in overall hit rates was included as random effect, because the estimation of more complex models did not reach convergence. Analogous to the response time analyses, we calculated the estimated probability of correct responses conditioned on all levels of the predictor variables as well as 95% confidence intervals as a measure of estimation accuracy.

## Results

Tables containing the full model output for all analyses can be found in the supplementary online material (Tables A1 and A2).

### Response times

As a service to the reader, we would like to review some important considerations when interpreting confidence intervals (see, e.g.,^35–37^, for more thorough discussions) before we describe our results. In short, the confidence intervals in Table 2 illustrate the ‘average’ estimated RTs and their precision, and they may be used for two inferential purposes: First, they can be used to judge whether the RTs differ significantly from zero (or any specific population value) at the 5% level of significance. All RTs displayed differed from zero as zero is not within the confidence limits. Notably, CIs can also be interpreted as a measure of evidence in the sense, that a population value of zero is less likely the further zero is outside the CI bounds. Second, these intervals may be used for between group comparisons, Cumming and Finch (2005) provide “rules of eye” for this objective: (1) The *p* value is roughly smaller than 0.05 when the overlap between CIs is less than 50%, (2) The *p* value is roughly smaller than 0.01 when CIs do not overlap. Furthermore, it is important to note that these rules *can* be misleading for within group comparisons (e.g., to compare standard and distractor RTs). This is due to the contribution of the correlation between repeated measures to the width of the CI. Therefore, it is important, to always consider the CIs for RT *differences* as well for inference purposes. The question whether the CI of a RT difference contains zero (‘non-significant’) or not (‘significant’) is equivalent to the respective statistical test at the 5% level. We hope that the use of CIs helps the reader to judge the evidence provided by our data in a meaningful unit (i.e. RT) rather than simply based on *p* values^38^.

#### Condition effect model

**Figure 2:**
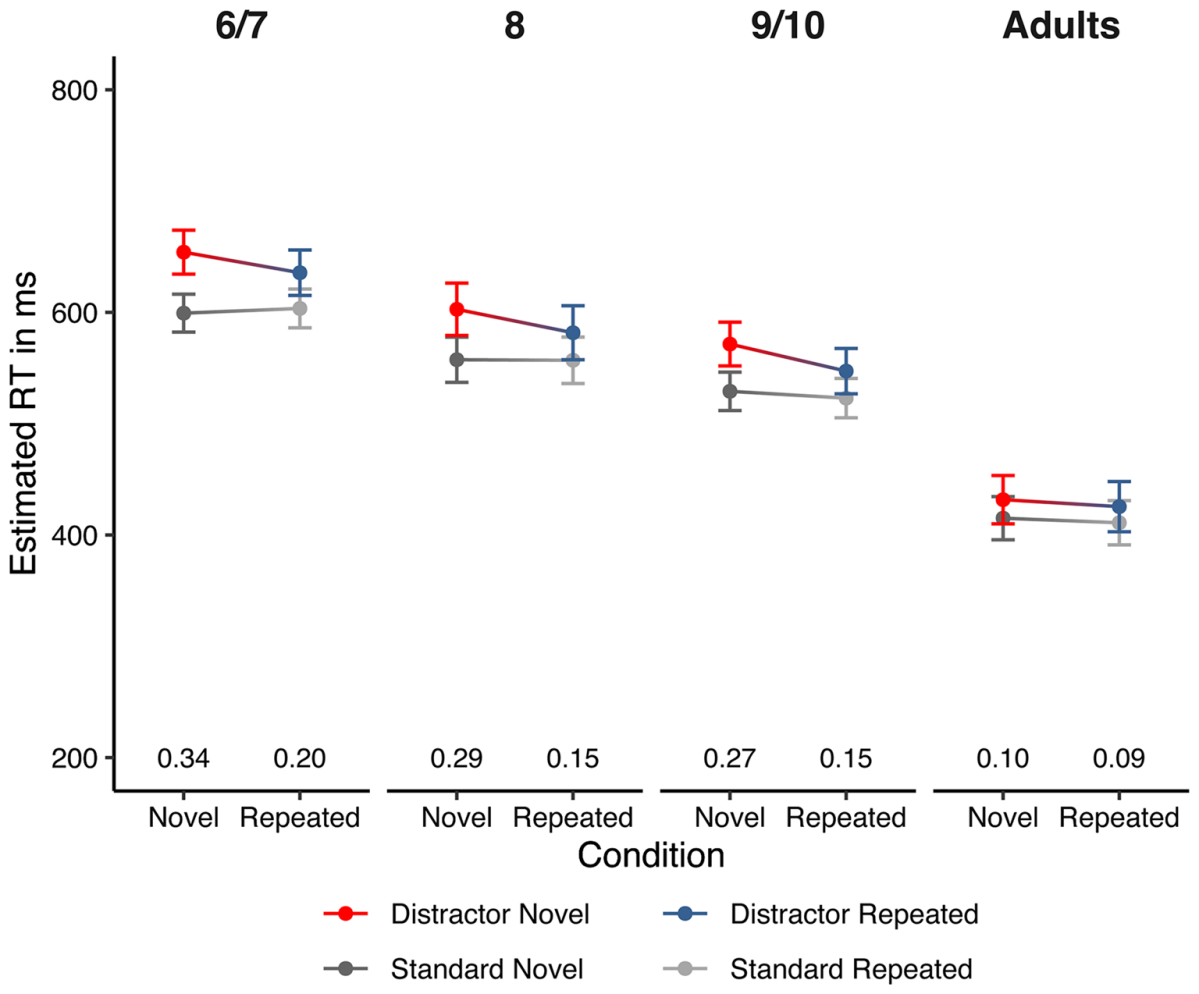
Model-implied response times as a function of sound, condition and age group. The error bars represent 95% confidence intervals. The numbers at the bottom are standardized distraction effects (i.e., $\widehat{RT}_{distractor} - \widehat{RT}_{standard}$ for *z*-standardized RTs).

The predictors (Sound, Condition, Age and their interactions) in the *condition effect model* explained a substantial amount of variation in the trial RTs ($R_{marginal}^{2}$ = 0.163) and all modeled effects represented a substantial amount of variation in the data ($R_{conditional}^{2}$ = 0.282). ANOVA-like model comparisons (Table 1) yielded a significant 3-way interaction of Sound, Condition and Age suggesting that the *combination* of all three predictors needs to be considered to explain the mean RTs. The estimated response times from the *condition effect model* for each condition and their confidence intervals are displayed in Fig. 2. These differences and their CIs are provided in Table 2 and indicate that without exception all age groups showed significant distraction effects in both novel and repeated condition.

**Table 1:** F-tests for main effects and interactions in the condition effect model.

	numDF	denDF	F-value	*p* value
(Intercept)	1	26,077	14,743.85	**<** **0.001**
Condition	1	26,077	13.63	**<** **0.001**
Sound	1	26,077	173.13	**<** **0.001**
Age	3	175	82.63	**<** **0.001**
Condition:sound	1	26,077	15.54	**<** **0.001**
Condition:age	3	26,077	0.68	0.562
Sound:age	3	26,077	6.62	**<** **0.001**
Condition:sound:age	3	26,077	3.08	**0.026**

Statistically significant results are marked in bold.

**Table 2:** Model-implied RTs and distraction effects.

Condition	Sound	6–7 years	8 years	9–10 years	Adults
Novel	Novel	654.11 [634.33; 673.88]	602.77 [579.26; 626.28]	571.53 [551.88; 591.19]	431.73 [410.00; 453.47]
	Standard	599.32 [582.29; 616.34]	557.48 [537.12; 577.83]	529.09 [511.83; 546.34]	415.11 [395.74; 434.49]
	Difference	**54.79** [41.24; 68.34]	**45.30** [29.76; 60.83]	**42.45** [30.33; 54.57]	**16.62** [4.73; 28.51]
Repeated	Repeated	635.64 [615.24; 656.05]	581.72 [557.46; 605.98]	547.25 [526.86; 567.64]	425.50 [402.92; 448.09]
	Standard	603.55 [586.13; 620.97]	556.98 [536.11; 577.84]	522.96 [505.26; 540.65]	411.00 [391.11; 430.89]
	Difference	**32.09** [18.59; 45.59]	**24.74** [9.27; 40.21]	**24.29** [12.11; 36.47]	**14.50** [2.58; 26.42]

The values in the table represent the predicted (or model-implied) RTs and distractions effects (i.e., differences distractor RT–standard RT).

These may be interpreted as if they were mean RT for the respective factor combination.

The main advantage of the LMM in this context is that it easily provides 95% confidence intervals (provided in square brackets) that take the repeated measures structure of the data and the heteroscedasticity between groups into account.

All groups showed significant distraction effects the novel and repeated condition (marked in bold).

In general, RTs decreased with age and RTs were slower for distractor than for standard sounds. Distraction effects varied as a function of age (Table 3). Especially in the novel condition, children showed larger distraction effects than adults (Fig. 2, for detailed results see Tables 2 and 3). Model-implied distraction effects of adults in the novel condition were 38.17 ms less than distraction effects of the youngest group, 28.71 ms less than those of the 8-year-olds, and 25.83 ms less than the oldest children (Table 3). All comparisons revealed significant differences since the CIs of the differences did not contain zero. In the repeated condition, only the youngest group differed tendentially from adults (although zero—strictly speaking—was within the CI bounds; Fig. 2, Table 3).

**Table 3:** Group differences in distraction effects in the condition effect model.

	6/7	8	9/10	Adults
6/7	–	*−* *9.49* *[−* *30.11, 11.12]*	*−* *12.34* *[−* *30.52, 5.84]*	***−*** ***38.17*** *[−* *56.20, −* *20.14]*
8	7.35 [− 13.18, 27.88]	–	*−* *2.85* *[−* *22.55, 16.86]*	***−*** ***28.71*** *[−* *48.24, −* *9.11]*
9/10	7.80 [− 10.38, 25.98]	0.45 [− 19.24, 20.14]	–	***−*** ***25.83*** *[−* *42.81; −* *8.85]*
Adults	***17.59*** [− 0.42, 35.60]	10.24 [− 9.29, 29.77]	9.79 [− 7.25, 26.83]	–

The values represent differences in distraction effects (i.e., differences between the groups in the quantity $\widehat{{{\text{RT}}}}_{{{\text{distractor}}}} - \widehat{{{\text{RT}}}}_{{{\text{standard}}}}$).

The upper triangle (italic) displays the differences between groups in the novel condition.

The lower triangle (underline) displays the differences in the repeated condition.

95% confidence intervals are given in square brackets.

A negative value indicates a smaller distraction effect in the group indicated by the column than in the group indicated by the row.

Substantial group differences (marked in bold) were observed for all children groups versus adults in the novel condition and for the youngest children versus adults in the repeated condition since CIs for the differences did not or did hardly cross zero.

The differences in the distraction effects between conditions were similar for all children groups, that is, in the youngest group distraction effects in the novel condition were 22.70 ms (CI 41.42, 3.97) larger than in the repeated condition, in the 8-year-olds distraction effects were 20.56 ms (CI 41.97, − 0.86) larger, in the 9–10-year-olds 18.16 ms (CI 34.86, 1.45) larger while in adults distraction effects differed only 2.12 ms (CI 18.30, − 14.07) between conditions. The CIs for the differences in children did not cross zero for the 6-year and 9–10-year-olds and only slightly crossed zero for the 8-year-olds. To sum up, distraction effects of all children groups in the novel condition substantially differ from those of adults. For children but not for adults, we observed substantially higher distraction effects in the novel condition than in the repeated condition.

**Figure 3:**
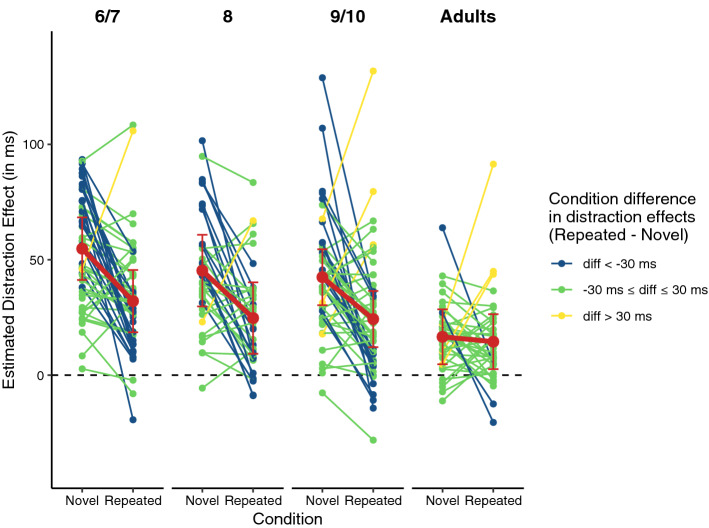
Model-implied distraction effects (i.e., $\Delta \widehat{{{\text{RT}}}} = \widehat{{{\text{RT}}}}_{{{\text{distractor}}}} - \widehat{{{\text{RT}}}}_{{{\text{standard}}}}$) as a function of condition and age group. The bold red lines represent the fixed effects (i.e., the ‘average’ distraction effect across participants). Error bars represent 95% confidence intervals. The thinner lines represent the distraction effects of individual participants. The color code reflects size and direction of the difference in the distraction effects between the novel and the repeated condition (i.e., $\Delta \widehat{{{\text{RT}}}}_{{{\text{repeated}}}} - \Delta \widehat{{{\text{RT}}}}_{{{\text{novel}}}}$).

Distraction effects occurred with remarkable consistency across participants. The estimated distraction effects (RT_Distractor_ − RT_Standard_), both aggregated across participants and for individual participants, are displayed in Fig. 3. A positive distraction effect (i.e., difference RT_Distractor_ − RT_Standard_) was observed in 172 out of 179 participants in the novel condition, and for 158 out of 179 participants in the repeated condition (see also Fig. 3 for an illustration). That is, with only few exceptions, all participants responded slower when a distractor sound was presented, compared to when a standard sound was presented in all conditions. The difference between distraction effects in the novel and repeated condition was less consistent across participants. Descriptively, with increasing age, less children showed higher distraction effects in the novel condition than in the repeated condition (6–7 years: 83%; 8 years: 70%; 9/10 years: 68%). In the adult group, no substantial differences between the distraction effects in the novel and repeated condition could be observed—and only a weak trend was observable across individuals (63% with higher distraction effects in the novel condition).

In line with the low amount of explained variance, we observed considerable residual variability between trials. That is, beyond the explained effects, single trials tended to vary extensively in their response times around the predicted values. Remarkably, age groups differed in trial-level variability with 6–7-year old children showing the greatest variability (*SD*_*Residual*_ = 153.82 ms) followed by 8-year-olds (*SD*_*Residual*_ = 139.67 ms), and 9–10-year-olds (*SD*_*Residual*_ = 118.61 ms). Adults showed substantially less variability across trials (*SD*_*Residual*_ = 78.30 ms).

#### Block effect model

The fit of the *block effect model* (in which we tested the change of the distraction effects over blocks in the experimental session) was similar to the *condition effect model*. The predictors *Age*, *Sound*, *Block, Randomization* and their interactions explained a substantial amount of variation in the trial RTs ($R_{marginal}^{2}$ = 0.172) and all modeled effects represented a substantial amount of variation in the data ($R_{conditional}^{2}$ = 0.282). We found several statistically significant 3-way interactions between the predictors, *F*_Sound × Block × Age_ (15, 26,001) = 5.13, *p* < 0.001; *F*_Sound × Block × Randomization_ (5, 26,001) = 4.80, *p* < 0.001; *F*_Sound × Age × Randomization_ (3, 26,001) = 2.67, *p* = 0.046. These findings support the notion that distraction effects changed as a function of *Randomization* and *Block* and that this also differed between age groups (Supplement Table A3 for all effect tests).

The estimated standard and distractor RTs from the *block effect model* are displayed as a function of sound, age group, randomization and block number in Supplement Figure A3. The same general patterns are observable as in the condition effects model. That is, overall RTs and differences between distractor and standard sounds tended to decrease with age. Within each age group, RTs, when a standard sound was presented, remained relatively unchanged across all blocks, irrespective of condition and randomization. However, especially for children, responses to the visual task when a distractor sound was presented, were slower in earlier blocks, but this effect decreased over time, most prominently for children starting with the novel condition.

**Figure 4:**
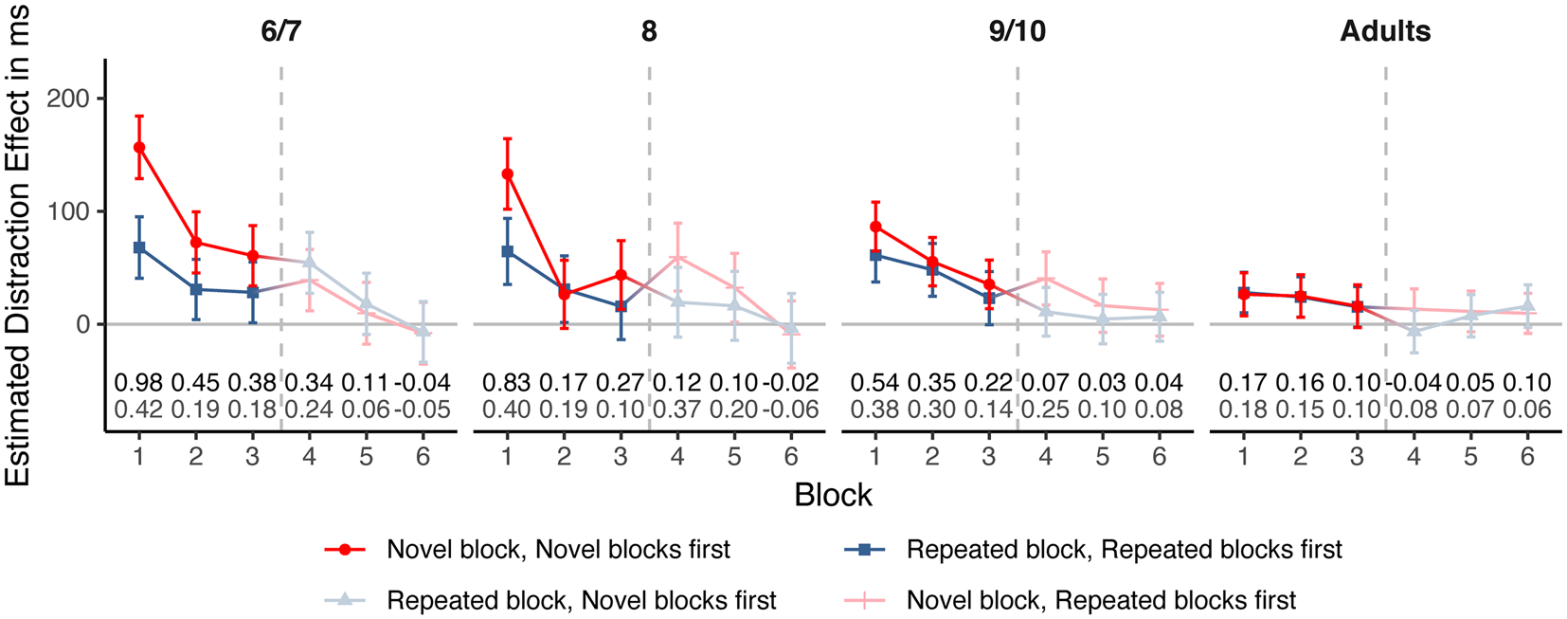
Model-implied distraction effects (i.e., $\Delta \widehat{{{\text{RT}}}} = \widehat{{{\text{RT}}}}_{{{\text{distractor}}}} - \widehat{{{\text{RT}}}}_{{{\text{standard}}}}$) from the block effect model as a function of block, randomization and age group. Error bars represent 95% confidence intervals. The numbers at the bottom are the standardized distraction effects (i.e. $\widehat{RT}_{distractor} - \widehat{RT}_{standard}$ for *z*-standardized RTs) when the novel blocks were presented first (black) and when the repeated blocks were presented first (grey). See Tables 4 and 5 for estimates and CIs as well as group comparisons.

A more in-depth analysis of this pattern is presented in Fig. 4 which depicts the distraction effects as a function of block, age and randomization. All children groups showed larger distraction effects than adults in the first block—irrespective of which condition was presented first—and no substantial group differences occurred in the last block.

Table 5 presents more details on estimates and confidence intervals. In the first block, distraction effects in children who started with the novel condition, were significantly increased compared to children who started with the repeated condition in the youngest age groups. The differences in distraction effects between the first block of the novel and repeated condition (Novel–Repeated) were 88.69 ms for 6–7-years-olds (CI 49.83, 127.54), 68.55 ms for 8-years-olds (CI 25.81, 111.29), 25.28 ms for 9–10-years-olds (CI − 6.78, 57.34), and − 1.59 ms for adults (CI − 27.81, 24.63). When the novel condition was presented first, distraction effects were significantly larger for the first block than for all remaining blocks in all children groups (Table 5, Supplement Table A4). In adults, only the 4th block (i.e., the first block after the change of conditions) was significantly different from the first block. All children but not adults, showed significantly reduced distraction effects from the first to the last (sixth) block irrespective whether the novel (1st vs. 6th block difference CIs 6–7 years: 126.70, 200.02; 8 years: 95.46, 177.93; 9 years: 51.67, 107.87) or the repeated condition was presented first (1st vs. 6th block difference CIs 6–7 years: 39.20, 112.56; 8 years: 34.41, 112.66; 9 years: 17.70, 78.83). When the repeated condition was presented first, the distraction effect in the first block was also descriptively higher than in the following blocks in children, but this pattern was less pronounced (significant differences are marked in bold italic in Table 4 and also in Table 5 for the reader’s convenience). For adults, no significant decline of distraction effects was observed across blocks and randomizations (1st vs. 6th block difference CIs novel first: − 12.02, 32.79; repeated first: − 2.60, 39.41).

**Table 4:** Group differences in distraction effects in the block effect model as a function of block and randomization.

Age	Randomization	Versus 6–7	Versus 8	Versus 9	Versus Adults
6–7	Novel first	–	*−* *23.55* *[−* *65.24, 18.13]*	*−* *70.19* *[−* *105.33, −* *35.04]*	***−*** ***130.12*** *[−* *163.79, −* *96.46]*
6–7	Repeated first	–	*−* *3.42* *[−* *43.4, 36.57]*	*−* *6.79* *[−* *42.88, 29.31]*	***−*** ***39.85*** *[−* *72.47, −* *7.23]*
8	Novel first	− 3.12 [− 44.00, 37.77]	–	*−* *46.63* *[−* *84.55, −* *8.72]*	***−*** ***106.57*** *[−* *143.13, −* *70.02]*
8	Repeated first	1.07 [− 39.33, 41.48]	–	*−* *3.37* *[−* *41.01, 34.27]*	***−*** ***36.44*** *[−* *70.76, −* *2.11]*
9	Novel first	− 13.40 [− 47.91, 21.1]	− 10.29 [− 47.94, 27.37]	–	***−*** ***59.94*** *[−* *88.81, −* *31.07]*
9	Repeated first	− 20.83 [− 56.82, 15.17]	− 21.90 [− 59.69, 15.89]	–	***−*** ***33.07*** *[−* *62.77, −* *3.37]*
Adults	Novel first	− 22.85 [− 55.69, 9.98]	− 19.73 [− 55.86, 16.40]	− 9.45 [− 38.16, 19.26]	–
Adults	Repeated first	− 17.63 [− 50.29, 15.04]	− 18.7 [− 53.34, 15.94]	3.20 [− 26.17, 32.57]	–

The values represent differences in distraction effects (i.e., differences between the groups in the quantity $\widehat{{{\text{RT}}}}_{{{\text{distractor}}}} - \widehat{{{\text{RT}}}}_{{{\text{standard}}}}$).

The upper triangle (italic) displays the differences between groups in the first block of the experiment. Substantial differences between children and adults are marked in bold italic (CIs did not cross zero).

The lower triangle (underline) displays the differences in last block of the experiment.

95% confidence intervals are given in square brackets.

A negative value indicates a smaller distraction effect in the group indicated by the column than in the group indicated by the row.

**Table 5:** Distraction effects in each block and for each randomization separately for all age groups.

Block	Randomization	6/7	8	9/10	Adults
*1 (Novel)*	*Novel first*	156.63* *[128.92, 184.33]*	133.08* *[101.93, 164.22]*	*86.44* *[64.82, 108.06]*	*26.51* *[7.38, 45.63]*
2 (Novel)	Novel first	**72.41** [45.36, 99.46]	**26.45** [− 3.76, 56.65]	**55.39** [33.94, 76.83]	24.98 [6.12, 43.84]
3 (Novel)	Novel first	**60.72** [34.05, 87.39]	**43.62** [13.16, 74.08]	**35.32** [13.76, 56.89]	16.04 [− 2.88, 34.97]
4 (Repeated)	Novel first	**54.4** [27.38, 81.41]	**19.52** [− 11.36, 50.4]	**11.02** [− 10.48, 32.51]	**-6.48** [− 25.23, 12.28]
5 (Repeated)	Novel first	**18.12** [− 8.95, 45.2]	**16.32** [− 14.2, 46.84]	**4.59** [− 17.28, 26.45]	7.61 [− 11.17, 26.4]
6 (Repeated)	Novel first	**-6.73** [− 33.61, 20.14]	**-3.62** [− 34.43, 27.2]	**6.67** [− 14.97, 28.31]	16.12 [− 2.75, 34.98]
1 (Repeated)	Repeated first	67.94* [40.7, 95.19]	64.53* [35.26, 93.79]	61.16 [37.48, 84.83]	28.09 [10.16, 46.03]
2 (Repeated)	Repeated first	**30.83** [4.16, 57.51]	31.06 [1.55, 60.56]	48.18 [24.77, 71.59]	24.16 [6.35, 41.97]
3 (Repeated)	Repeated first	**28.25** [1.35, 55.14]	**15.91** [− 13.5, 45.32]	**23.14** [− 0.35, 46.64]	15.46 [− 2.44, 33.36]
4 (Novel)	Repeated first	39.09 [11.99, 66.2]	59.49 [29.46, 89.51]	40.68 [17.17, 64.19]	13.42 [− 4.47, 31.31]
5 (Novel)	Repeated first	**9.74** [− 17.61, 37.09]	32.36 [1.95, 62.77]	**16.49** [− 7.1, 40.07]	11.39 [− 6.6, 29.39]
6 (Novel)	Repeated first	**-7.94** [− 35.32, 19.45]	**-9.01** [− 38.72, 20.7]	**12.89** [− 10.47, 36.25]	9.69 [− 8.12, 27.5]

The values are distraction effects (i.e., $\widehat{{{\text{RT}}}}_{{{\text{distractor}}}} - \widehat{{{\text{RT}}}}_{{{\text{standard}}}}$) in the respective combination of group, block and randomization.

Positive values indicate slower responses in the presence of distractor sounds.

95% confidence intervals are given in square brackets.

Italic marked line highlights the first novel block when the novel condition was presented first, underline highlights the first repeated block when the repeated condition was presented first.

Asterisks mark significantly increased distraction effects in the novel condition compared to the repeated condition in the first block in both youngest age groups (non-overlapping CIs of the respective differences: CIs [49.83, 127.54] for 6–7-years-olds and [25.81, 111.29] for 8-years-olds).

Bold print marks blocks that significantly differ from the respective first block (on the basis of CIs of difference values in Table A4 for which CIs did not overlap with zero).

In sum, distraction effects in the first block were larger in children than in adults, while no substantial group differences were observed in the last block. The order of conditions modulated the distraction effects differently in the different age groups. When the novel condition was presented first, all children groups (but not adults) showed increased distraction effects in the first block compared with following blocks. In addition, children aged 6–8 years were significantly more distracted by novel sounds than by repeated sounds during the first block.

Similar to the condition effects model, we observed considerable residual variability between trials. Age groups differed in trial-level variability with 6–7-year old children showing the greatest variability (*SD*_*Residual*_ = 154.08 ms) followed by 8-year-olds (*SD*_*Residual*_ = 140.00 ms), and 9–10-year-olds (*SD*_*Residual*_ = 120.09 ms). Adults showed substantially less variability across trials (*SD*_*Residual*_ = 78.93 ms).

### Hit rates

Across all conditions and groups, the estimated probability of correct responses was very high (*M* = 0.96, *SD* = 0.02). Overall, there was a slight tendency toward more correct responses in the repeated condition and adults were slightly more likely to give a correct response in the novel condition than children (Supplement Figure A1). Most importantly, no significant differences in the estimated probabilities were found between standards and distractor sounds, except for the adult group, where correct responses were slightly more likely when a distractor was presented in the repeated condition. That is, no distraction effects were observed. No distraction effects in the probability of correct responses were found in the block-wise analysis and there were no differences in the probability of correct responses between experimental blocks (Supplement Figure A2).

## Discussion

Children, but not adults, showed large distraction effects in the first block(s), particularly in response to novels, that declined to an adults' level in the last block(s). New or repeated distractor sounds increased RTs in a visual categorization task more so in children than in adults. Novel sounds caused larger distraction effects than repeated distractor sounds in children only.

Unexpected and task-irrelevant distractor sounds impaired performance in children and adults. This distraction effect was observed in almost all participants (94% in the novel condition; 88% in the repeated condition). Following current models on distraction of attention^5,6^, resources spent on orienting, evaluation of distractor sounds, and reorienting back to the task are no longer available for task-related processes, resulting in prolonged RTs. Children's distraction effects were generally larger than those of adults. This was evident for novel distractor sounds. Although, strictly speaking, the statistical analyses only support this claim for the novel condition, qualitatively, the same trends occurred in the repeated condition. We tend to conclude that the evidence for the decay was simply weaker due to the smaller initial distraction effects. Stronger distraction effects in children compared to adults are in line with the majority of studies and with the developmental trajectory of involved brain networks. This continuing maturation of attention control needs to be discussed considering the impact of a sounds' novelty on age differences in distraction effects. Novel compared to repeated distractor sounds caused significantly increased distraction effects in children but not in adults. The lack of a condition effect in adults is compatible to a similar study reporting comparable distraction effects to uniquely and repeatedly presented distractor sounds in adults^39^. The increased novel-related distraction effect was observed in all groups of children and in the majority of individuals. Although only descriptively, the number of individuals showing a stronger distraction effect in response to novel compared to repeated distractor sounds decreased with age. Most of the youngest children (80%), but only 66% of the oldest children showed this novelty effect.

However, all these age- and condition-related differences in mean distraction effects have to be interpreted considering the time course of distraction effects throughout the session. In children, distraction effects were maximal in the first block and declined with the number of presented experimental blocks, particularly when the novel condition was presented first. Such a decline was not observed in adults. Importantly, the decline of distraction effects was exclusively due to a decline of RTs for distractor sounds, implying that the observed decline was specific and not due to differences in general sound processing throughout the session. All children groups consistently differed from adults in the first presented block, and these differences could not be explained by generally faster responses resulting in proportionally lower distraction effects (see Supplement part D). This age effect for the first block was observed regardless whether the novel or repeated condition was presented first. In both the youngest age groups, distraction effects in the first block were significantly larger when presenting the novel condition first, than when presenting the repeated condition first. When presenting the repeated condition first, nevertheless, a small increase of distraction effects could be observed in the first novel block (i.e., the fourth experimental block) in children. These patterns demonstrate that novel sounds initially have a particularly strong impact on performance in children that decreases with age. In conclusion, our results indicate that the initial susceptibility to unexpected novel sounds decreases throughout middle childhood.

The decline of distraction effects throughout the experiment indicates that short-term learning processes enhanced attention control, that is children were better able to reduce impairing effects of novel sounds on performance. While we observed a strong impact of novelty on initial distraction in children, distraction effects in the last block were on a similar level in all age groups. The similar distraction effects in the last presented block in children and adults indicates remarkable attention control abilities, even in the youngest children, as long as they have the possibility to perform a sufficient number of trials. In the framework of the three-stage model of involuntary attention [e.g.,^5,6^], children might allocate more resources for involuntary orienting toward the novel sounds, for their evaluation and for voluntary reorienting of attention back to task at hand during the first block. This results in stronger attentional distraction. It has been shown previously that the different stages of the distraction process follow different developmental time courses^21^ and continue to develop into adolescence^40^. We assume that children learn fast to limit the impact of novel irrelevant information by stopping extended evaluation and efficiently re-orient to the main task. On a cognitive level, inhibitory mechanisms could interact with short-term learning. With an increasing number of novel sounds, children might learn to categorize novel sounds as task-irrelevant and to inhibit further evaluation. Inhibitory control of irrelevant information develops throughout childhood and has been linked to the maturation of the frontal cortex [for review see^41^]. Inhibition mechanisms could take place on several levels of the distraction process (e.g., inhibition of attentional orienting, inhibition of behavioral responses) and might be accessible to short-term learning with a different time course in different age groups. In addition to these learning processes, we assume that age-sensitive strategic processes involved in self-regulation and the maturity level of voluntary attention processes contribute to distraction effects.

From a functional perspective, increased initial distraction effects in younger children could be influenced by the relevance of novel events for their acquisition of knowledge about the world. New events have to be detected before evaluation and integration in the long-term memory. This can be supported by a less focused and more exploratory behavior, resulting in increased distractibility. On the level of neuronal networks, such an exploratory behavior has been linked to increased tonic activity of the noradrenergic locus-coeruleus norepinephrine (LC-NE) system [for review see^42^]. Recent studies pronounced the role of the LC-NE system for attention networks^43^, particularly in the context of novel events^4,44^. In addition, it has been assumed that distraction effects are a sum of costs of orienting of attention toward novel sounds and benefits of increased arousal (that is strongly modulated by LC activity) in response to novel sounds^45–47^. The underlying mechanisms might develop with different time courses. We hypothesize that these mechanisms are partly controlled by learning processes.

In sum, different cognitive functions can contribute to the fast learning of distraction control. Future developmental studies could address the precise nature of orienting and evaluation and inhibition mechanisms during distraction of attention, their neuronal basis including the link between involuntary attention and arousal modulated by LC-NE system and the influence of strategies. A limitation of the present study is, that we did not test the generalizability of the observed learning effects, that is, do children show reduced distraction effects when testing them again later or do they learn even faster to successfully control their attention?

## Conclusion

We demonstrated that children were more distracted by unexpected and task-irrelevant novel sounds than adults. All children groups were more distracted by novel than by repeated sounds while adults were similarly distracted by novel and repeated sounds. Especially younger children were initially highly susceptible to novel sounds, but distraction effects disproportionately decreased with the number of presented sounds, indicating an interaction of distraction effects with short-term learning processes.

The age-related impact of short-term learning processes on attention control has important implications for further research. First, results demand for the analysis of timelines of distraction effects in addition to mean values in developmental studies as averaging may distort results. This might partly explain the inconsistent findings on distractibility in middle childhood, because experiments differ in the number of presented sounds. Second, attention control is modulated by short-term learning processes. We discussed two models on the neuronal and cognitive level, that have to be further specified and integrated in developmental attention models. Third, in everyday life, distractor sounds occur randomly and uniquely. This results in attentional distraction, which corresponds to the distraction effects observed in the first novel block, that significantly differ between age groups. Consequently, effects of attentional distraction in real-life learning environments are probably much stronger than estimated by previous studies. On the other hand, if children have the opportunity to learn that distractors can occur in a certain context, then they are able to successfully control attention better than assumed. An enhanced understanding of the developmental trajectory of attention control in the presence of distracting events and the specification of involved learning processes is crucial to understand and treat attention disorders and to provide optimal learning environments for children. The design of such learning environments can take into account age-related initial strong distraction by unexpected novel events and exploit fast learning of attention control.

## Supplementary Information

Supplementary Information.

## Data Availability

Interested researchers can access raw data on zenodo.org (https://doi.org/10.5281/zenodo.4121860).
