# Supplementary material for: Distraction of attention by novel sounds in children declines fast
Source: Sci Rep. 2021 Mar 5;11:5308. doi: 10.1038/s41598-021-83528-y (PMC7935912; doi:10.1038/s41598-021-83528-y)
Supplement: Supplementary file 1 — Supplementary Information. [file 41598_2021_83528_MOESM1_ESM.pdf]

Supplement

Distraction of attention by novel sounds in children declines fast

Nicole Wetzel [1)2)3)], Andreas Widmann [1)4)], and Florian Scharf [5)]

[1)] Leibniz Institute for Neurobiology, Magdeburg, Germany

[2)] Center for Behavioral Brain Sciences Magdeburg, Germany

[3)] University of Applied Sciences Magdeburg-Stendal, Germany

[4)] Leipzig University, Germany

[5)] University of Münster, Germany

Part A – Supplemental Tables and Figures

Table A1

Estimates and Confidence Intervals of the Parameters for the Condition-Effect Model

Effect	Parameter	Estimate	CI (95%)	Comments
Fixed	Intercept	599.32	[582.29; 616.34]	Predicted RT to standard sounds in the novel condition for the youngest children
	Condition	4.23	[-7.54; 16.01]	Difference in standard RTs between conditions (Repeated – Novel) for the youngest children
	Sound	54.79	[41.24; 68.34]	Difference in RTs between Sounds (Distractor - Standard) in the novel condition for the youngest children
	Group 8	-41.84	[-68.56; -15.12]	Difference in standard RTs in the novel condition between groups (Age 8 – Age 6/7)
	Group 9/10	-70.23	[-94.63; -45.83]	Difference in standard RTs in the novel condition between groups (Age 9/10 – Age 6/7)
	Group Adults	-184.2	[-210.17; -158.23]	Difference in standard RTs in the novel condition between groups (Adults – Age 6/7)
	Condition x Sound	-22.70	[-41.42; -3.97]	Difference in Parameter “Sound” between conditions (Repeated – Novel) in the youngest children
	Condition x Group 8	-4.73	[-22.76; 13.29]	Difference in Parameter “Condition” between groups (Age 8 – Age 6/7)
	Condition x Group 9/10	-10.36	[-26.44; 5.71]	Difference in Parameter “Condition” between groups (Age 9/10 – Age 6/7)
	Condition x Group Adults	-8.35	[-24.59; 7.9]	Difference in Parameter “Condition” between groups (Adults– Age 6/7)
	Sound x Group 8	-9.49	[-30.11; 11.12]	Difference in Parameter “Sound” between groups (Age 8 – Age 6/7)
	Sound x Group 9/10	-12.34	[-30.52; 5.84]	Difference in Parameter “Sound” between groups (Age 9/10 – Age 6/7)
	Sound x Group Adults	-38.17	[-56.2; -20.14]	Difference in Parameter “Sound” between groups (Adults– Age 6/7)
	Condition x Sound x Group 8	2.14	[-26.31; 30.59]	Difference in Parameter “Condition x Sound” between groups (Age 8 – Age 6/7)
	Condition x Sound x Group 9/10	4.54	[-20.55; 29.63]	Difference in Parameter “Condition x Sound” between groups (Age 9/10 – Age 6/7)
	Condition x Sound x Group Adults	20.58	[-4.17; 45.33]	Difference in Parameter “Condition x Sound” between groups (Adults– Age 6/7)

		SD	*Correlations*			
Random	Intercept	60.00	1			
	Condition	31.76	-0.21	1		
	Sound	31.90	-0.06	0.23	1	
	Condition x Sound	42.81	0.02	-0.21	-0.67	1
	Residual	153.03	Intercept	Condition	Sound	Condition x Sound

Note. CI = 95% confidence interval. The dependent variable was RT (ms). Age (reference = 6/7), sound (standard = 0, distractor = 1) and condition (0 = novel, 1 = repeated) were dummy-coded predictors. The intercept reflects the estimated RT in the youngest age group for targets after distractors in the novel condition. Note that all results reported in the main

article can be computed from this model by simple recoding (i.e., changing the reference categories).

Table A2

Estimates and Confidence Intervals of the Parameters for the Block-Effect Model

Effect	Parameter	Estimate	CI (95%)	Comment
Fixed	Intercept	598.72	[572.25; 625.2]	Predicted RT to standard sounds in the first block for the youngest children starting with the novel condition
	Sound	156.63	[128.92; 184.33]	Difference in RTs between Sounds (Distractor - Standard) in the first block for the youngest children starting with the novel condition
	Block 2	-4.68	[-24.78; 15.42]	Difference in RTs to standard sounds between Blocks (2-1) for the youngest children starting with the novel condition
	Block 3	-7.76	[-27.89; 12.38]	Difference in RTs to standard sounds between Blocks (3-1) for the youngest children starting with the novel condition
	Block 4	-15.86	[-36.07; 4.35]	Difference in RTs to standard sounds between Blocks (4-1) for the youngest children starting with the novel condition
	Block 5	16.19	[-3.87; 36.26]	Difference in RTs to standard sounds between Blocks (5-1) for the youngest children starting with the novel condition
	Block 6	19.38	[-0.65; 39.4]	Difference in RTs to standard sounds between Blocks (6-1) for the youngest children starting with the novel condition
	Group 8	-50.62	[-92.18; -9.07]	Difference in standard RTs in the first block for participants starting with the novel condition between groups (Age 8 – Age 6/7)
	Group 9/10	-58.54	[-95.09; -22]	Difference in standard RTs in the first block for participants starting with the novel condition between groups (Age 9/10 – Age 6/7)
	Group Adults	-167.99	[-207.28; -128.69]	Difference in standard RTs in the first block for participants starting with the novel condition between groups (Adults – Age 6/7)
	Randomization	1.17	[-36.48; 38.82]	Difference in RTs to standard sounds between the youngest children starting with the novel condition and the youngest children starting with the repeated condition
	Sound x Block 2	-84.22	[-120.99; -47.44]	Difference in Parameter “Sound” between blocks (2 – 1) for the youngest children starting with the novel condition
	Sound x Block 3	-95.91	[-132.4; -59.42]	Difference in Parameter “Sound” between blocks (3 – 1) for the youngest children starting with the novel condition
	Sound x Block 4	-102.23	[-138.98; -65.49]	Difference in Parameter “Sound” between blocks (4 – 1) for the youngest children starting with the novel condition
	Sound x Block 5	-138.50	[-175.3; -101.7]	Difference in Parameter “Sound” between blocks (5 – 1) for the youngest children starting with the novel condition
	Sound x Block 6	-163.36	[-200.02; -126.7]	Difference in Parameter “Sound” between blocks (6 – 1) for the youngest children starting with the novel condition

Effect	Parameter	Estimate	CI (95%)	Comment
	Sound x Group 8	-23.55	[-65.24; 18.13]	Difference in Parameter "Sound" between groups (Age 8 – Age 6/7) for participants starting with the novel condition
	Sound x Group 9/10	-70.19	[-105.33; -35.04]	Difference in Parameter "Sound" between groups (Age 9/10 – Age 6/7) for participants starting with the novel condition
	Sound x Group Adults	-130.12	[-163.79; -96.46]	Difference in Parameter "Sound" between groups (Adults– Age 6/7) for participants starting with the novel condition
	Block 2 x Group 8	-0.54	[-30.58; 29.49]	Difference in Parameter "Block 2" between groups (Age 8 – Age 6/7) for participants starting with the novel condition
	Block 3 x Group 8	11.81	[-18.43; 42.05]	Difference in Parameter "Block 3" between groups (Age 8 – Age 6/7) for participants starting with the novel condition
	Block 4 x Group 8	14.27	[-16.06; 44.6]	Difference in Parameter "Block 4" between groups (Age 8 – Age 6/7) for participants starting with the novel condition
	Block 5 x Group 8	-18.51	[-48.83; 11.82]	Difference in Parameter "Block 5" between groups (Age 8 – Age 6/7) for participants starting with the novel condition
	Block 6 x Group 8	-9.50	[-39.59; 20.58]	Difference in Parameter "Block 6" between groups (Age 8 – Age 6/7) for participants starting with the novel condition
	Block 2 x Group 9/10	-13.23	[-38.6; 12.14]	Difference in Parameter "Block 2" between groups (Age 9/10 – Age 6/7) for participants starting with the novel condition
	Block 3 x Group 9/10	9.97	[-15.42; 35.36]	Difference in Parameter "Block 3" between groups (Age 9/10 – Age 6/7) for participants starting with the novel condition
	Block 4 x Group 9/10	8.70	[-16.74; 34.15]	Difference in Parameter "Block 4" between groups (Age 9/10 – Age 6/7) for participants starting with the novel condition
	Block 5 x Group 9/10	-25.35	[-50.7; 0]	Difference in Parameter "Block 5" between groups (Age 9/10 – Age 6/7) for participants starting with the novel condition
	Block 6 x Group 9/10	-32.97	[-58.31; -7.63]	Difference in Parameter "Block 6" between groups (Age 9/10 – Age 6/7) for participants starting with the novel condition
	Block 2 x Group Adults	-9.11	[-32.63; 14.4]	Difference in Parameter "Block 2" between groups (Adults – Age 6/7) for participants starting with the novel condition
	Block 3 x Group Adults	1.42	[-22.15; 24.99]	Difference in Parameter "Block 3" between groups (Adults – Age 6/7) for participants starting with the novel condition

Effect	Parameter	Estimate	CI (95%)	Comment
	Block 4 x Group Adults	6.70	[-16.96; 30.36]	Difference in Parameter “Block 4” between groups (Adults – Age 6/7) for participants starting with the novel condition
	Block 5 x Group Adults	-36.74	[-60.28; -13.2]	Difference in Parameter “Block 5” between groups (Adults – Age 6/7) for participants starting with the novel condition
	Block 6 x Group Adults	-38.58	[-62.06; -15.1]	Difference in Parameter “Block 6” between groups (Adults – Age 6/7) for participants starting with the novel condition
	Sound x Randomization	-88.68	[-127.54; -49.83]	Difference in Parameter “Sound” between Randomizations (Repeated first – novel first)
	Block 2 x Randomization	1.88	[-26.36; 30.12]	Difference in Parameter “Block 2” between Randomizations (Repeated first – novel first)
	Block 3 x Randomization	16.1	[-12.2; 44.41]	Difference in Parameter “Block 3” between Randomizations (Repeated first – novel first)
	Block 4 x Randomization	8.45	[-19.91; 36.81]	Difference in Parameter “Block 4” between Randomizations (Repeated first – novel first)
	Block 5 x Randomization	-4.23	[-32.64; 24.18]	Difference in Parameter “Block 5” between Randomizations (Repeated first – novel first)
	Block 6 x Randomization	-9.83	[-38.14; 18.48]	Difference in Parameter “Block 6” between Randomizations (Repeated first – novel first)
	Group 8 x Randomization	16.13	[-42.12; 74.38]	Difference in Parameter “Group 8” between Randomizations (Repeated first – novel first)
	Group 9/10 x Randomization	-27.73	[-80.47; 25]	Difference in Parameter “Group 9/10” between Randomizations (Repeated first – novel first)
	Group Adults x Randomization	-22.45	[-77.22; 32.33]	Difference in Parameter “Adults” between Randomizations (Repeated first – novel first)
	Sound x Block 2 x Group 8	-22.41	[-77.3; 32.49]	Difference in Parameter “Sound x Block 2” between groups (Age 8 – Age 6/7) for participants starting with the novel condition
	Sound x Block 3 x Group 8	6.45	[-48.4; 61.3]	Difference in Parameter “Sound x Block 3” between groups (Age 8 – Age 6/7) for participants starting with the novel condition
	Sound x Block 4 x Group 8	-11.32	[-66.58; 43.93]	Difference in Parameter “Sound x Block 4” between groups (Age 8 – Age 6/7) for participants starting with the novel condition
	Sound x Block 5 x Group 8	21.75	[-33.36; 76.85]	Difference in Parameter “Sound x Block 5” between groups (Age 8 – Age 6/7) for participants starting with the novel condition
	Sound x Block 6 x Group 8	26.67	[-28.5; 81.84]	Difference in Parameter “Sound x Block 6” between groups (Age 8 – Age 6/7) for participants starting with the novel condition

Effect	Parameter	Estimate	CI (95%)	Comment
	Sound x Block 2 x Group 9/10	53.17	[6.98; 99.35]	Difference in Parameter "Sound x Block 2" between groups (Age 9/10 – Age 6/7) for participants starting with the novel condition
	Sound x Block 3 x Group 9/10	44.79	[-1.22; 90.81]	Difference in Parameter "Sound x Block 3" between groups (Age 9/10 – Age 6/7) for participants starting with the novel condition
	Sound x Block 4 x Group 9/10	26.81	[-19.37; 73]	Difference in Parameter "Sound x Block 4" between groups (Age 9/10 – Age 6/7) for participants starting with the novel condition
	Sound x Block 5 x Group 9/10	56.65	[10.24; 103.05]	Difference in Parameter "Sound x Block 5" between groups (Age 9/10 – Age 6/7) for participants starting with the novel condition
	Sound x Block 6 x Group 9/10	83.59	[37.4; 129.78]	Difference in Parameter "Sound x Block 6" between groups (Age 9/10 – Age 6/7) for participants starting with the novel condition
	Sound x Block 2 x Group Adults	82.69	[39.63; 125.75]	Difference in Parameter "Sound x Block 2" between groups (Adults – Age 6/7) for participants starting with the novel condition
	Sound x Block 3 x Group Adults	85.45	[42.61; 128.29]	Difference in Parameter "Sound x Block 3" between groups (Adults – Age 6/7) for participants starting with the novel condition
	Sound x Block 4 x Group Adults	69.25	[26.26; 112.24]	Difference in Parameter "Sound x Block 4" between groups (Adults – Age 6/7) for participants starting with the novel condition
	Sound x Block 5 x Group Adults	119.62	[76.57; 162.66]	Difference in Parameter "Sound x Block 5" between groups (Adults – Age 6/7) for participants starting with the novel condition
	Sound x Block 6 x Group Adults	152.98	[110.01; 195.94]	Difference in Parameter "Sound x Block 6" between groups (Adults – Age 6/7) for participants starting with the novel condition
	Sound x Block 2 x Randomization	47.11	[-4.46; 98.68]	Differences between Randomizations in the respective lower order effects (Repeated first – novel first). *This also refers to the following lines.*
	Sound x Block 3 x Randomization	56.21	[4.73; 107.69]	
	Sound x Block 4 x Randomization	73.38	[21.61; 125.15]	
	Sound x Block 5 x Randomization	80.30	[28.36; 132.24]	
	Sound x Block 6 x Randomization	87.48	[35.63; 139.34]	
	Sound x Group 8 x Randomization	20.14	[-37.63; 77.9]	

Effect	Parameter	Estimate	CI (95%)	Comment
	Sound x Group 9/10 x Randomization	63.40	[13.02; 113.78]	
	Sound x Group Adults x Randomization	90.27	[43.4; 137.15]	
	Block 2 x Group 8 x Randomization	-5.21	[-47.23; 36.81]	
	Block 3 x Group 8 x Randomization	-16.62	[-58.89; 25.66]	
	Block 4 x Group 8 x Randomization	-14.45	[-56.69; 27.78]	
	Block 5 x Group 8 x Randomization	13.53	[-28.98; 56.03]	
	Block 6 x Group 8 x Randomization	5.43	[-36.8; 47.66]	
	Block 2 x Group 9/10 x Randomization	6.56	[-29.82; 42.95]	
	Block 3 x Group 9/10 x Randomization	-6.93	[-43.36; 29.51]	
	Block 4 x Group 9/10 x Randomization	2.69	[-33.76; 39.15]	
	Block 5 x Group 9/10 x Randomization	24.20	[-12.3; 60.69]	
	Block 6 x Group 9/10 x Randomization	34.30	[-2.15; 70.75]	
	Block 2 x Group Adults x Randomization	11.90	[-20.97; 44.77]	
	Block 3 x Group Adults x Randomization	-14.78	[-47.74; 18.19]	
	Block 4 x Group Adults x Randomization	6.08	[-26.94; 39.1]	
	Block 5 x Group Adults x Randomization	19.46	[-13.61; 52.54]	
	Block 6 x Group Adults x Randomization	22.20	[-10.74; 55.14]	

Effect	Parameter	Estimate	CI (95%)	Comment
	Sound x Block 2 x Group 8 x Randomization	26.05	[-50.37; 102.46]	
	Sound x Block 3 x Group 8 x Randomization	-15.37	[-91.8; 61.05]	
	Sound x Block 4 x Group 8 x Randomization	35.13	[-41.89; 112.16]	
	Sound x Block 5 x Group 8 x Randomization	4.29	[-72.87; 81.45]	
	Sound x Block 6 x Group 8 x Randomization	-24.33	[-101.27; 52.62]	
	Sound x Block 2 x Group 9/10 x Randomization	-29.03	[-95.19; 37.13]	
	Sound x Block 3 x Group 9/10 x Randomization	-43.11	[-109.26; 23.05]	
	Sound x Block 4 x Group 9/10 x Randomization	-18.44	[-84.8; 47.93]	
	Sound x Block 5 x Group 9/10 x Randomization	-43.12	[-109.77; 23.53]	
	Sound x Block 6 x Group 9/10 x Randomization	-55.98	[-122.41; 10.45]	
	Sound x Block 2 x Group Adults x Randomization	-49.52	[-109.54; 10.5]	
	Sound x Block 3 x Group Adults x Randomization	-58.38	[-118.37; 1.61]	
	Sound x Block 4 x Group Adults x Randomization	-55.07	[-115.25; 5.11]	
	Sound x Block 5 x Group Adults x Randomization	-78.11	[-138.48; -17.73]	
	Sound x Block 6 x Group Adults x Randomization	-95.50	[-155.77; -35.23]	
		SD	*Correlations*	
Random	Intercept	59.13	1	
	Sound	22.68	-0.002	1
	Residual	153.14	Intercept	Sound

Note. CI = 95% confidence interval. The dependent variable was RT (ms). Age (reference = 6/7), sound (standard = 0, distractor = 1), block (reference = first block) and randomization (reference = novel condition first) were dummy-coded predictors. For instance, the intercept reflects the estimated RT in the youngest age group for targets after distractors in the first block and for children who were first presented with the novel condition. Note that all results reported in the main article can be computed from this model by simple recoding (i.e., changing the reference categories). Exemplary comments are added in the right column.

Table A3

F-tests for main effects and interactions in the block effect model

	numDF	denDF	F-value	p-value
(Intercept)	1	26001	14136.73	**<0.001**
Sound	1	26001	17.21	**<0.001**
Block	5	26001	17.63	**<0.001**
Age	3	171	79.19	**<0.001**
Randomization	1	171	0.21	0.651
Sound:Block	5	26001	28.51	**<0.001**
Sound:Age	3	26001	6.56	**<0.001**
Block:Age	15	26001	1.94	**0.016**
Sound:Randomization	1	26001	2.37	0.124
Block:Randomization	5	26001	6.83	**<0.001**
Group:Randomization	3	171	0.50	0.685
Sound:Block:Age	15	26001	5.13	**<0.001**
Sound:Block:Randomization	5	26001	4.80	**<0.001**
Sound:Age:Randomization	3	26001	2.67	**0.046**
Block:Age:Randomization	15	26001	0.98	0.472
Sound:Block:Age:Randomization	15	26001	1.28	0.208

Note. Statistically significant results are marked in bold.

Table A4

Differences in distraction effects between the first and the remaining blocks in the block effect model

Age	Randomization	vs Block 2	vs Block 3	vs Block 4	vs Block 5	vs Block 6
6-7	Novel first	**84.22**	**95.91**	**102.23**	**138.5**	**163.36**
		[47.44, 120.99]	[59.42, 132.4]	[65.49, 138.98]	[101.7, 175.3]	[126.7, 200.02]
8	Novel first	**106.63**	**89.46**	**113.56**	**116.76**	**136.69**
		[65.87, 147.38]	[48.51, 130.41]	[72.29, 154.82]	[75.74, 157.77]	[95.46, 177.93]
9	Novel first	**31.05**	**51.12**	**75.42**	**81.85**	**79.77**
		[3.11, 59]	[23.08, 79.15]	[47.44, 103.4]	[53.58, 110.13]	[51.67, 107.87]
Adults	Novel first	1.52	10.46	**32.98**	18.89	10.39
		[-20.88, 23.93]	[-11.99, 32.91]	[10.66, 55.29]	[-3.45, 41.23]	[-12.02, 32.79]
6-7	Repeated first	**37.11**	**39.7**	28.85	**58.21**	**75.88**
		[0.95, 73.26]	[3.38, 76.01]	[-7.62, 65.32]	[21.54, 94.87]	[39.2, 112.56]
8	Repeated first	33.47	**48.62**	5.04	32.17	**73.54**
		[-5.5, 72.44]	[9.72, 87.52]	[-34.32, 44.40]	[-7.49, 71.82]	[34.41, 112.66]
9	Repeated first	12.98	**38.01**	20.48	**44.67**	**48.27**
		[-17.63, 43.58]	[7.35, 68.68]	[-10.2, 51.16]	[13.93, 75.41]	[17.7, 78.83]
Adults	Repeated first	3.93	12.63	14.67	16.7	18.4
		[-17.07, 24.93]	[-8.45, 33.71]	[-6.4, 35.74]	[-4.46, 37.85]	[-2.6, 39.41]

Note. The values are differences in distraction effects (i.e., block 1 minus respective block) in the respective combination of group, block and randomization. Positive values indicate higher distraction effects in the first versus the respective block. For instance, the distraction effect in the first block for 6–7-year-olds who started with the novel condition was 84.22 ms higher than in the second block. 95% confidence intervals are given in square brackets. Bold numbers indicate significantly increased distraction effects in the first versus the respective following block (CIs not-overlapping with zero in the respective fields). Please note that these blocks were also highlighted in bold in table 7 for the reader's convenience.

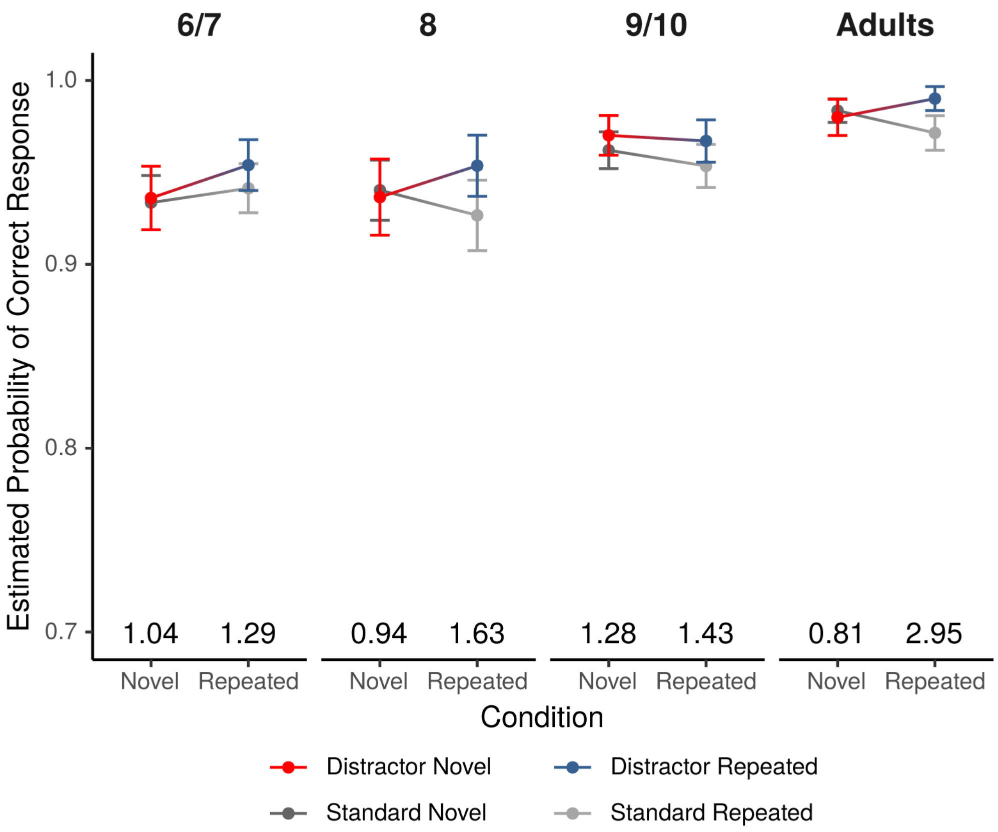

Figure A1.
Model-Implied Hit Rate of the Condition-Effect Model as a Function of Condition (novel versus repeated), Age, and Sound Type Note. Error bars represent 95% confidence intervals. The numbers at the bottom are odds ratios for distraction effects (i.e. $\frac{Odds_{distractor}}{Odds_{standard}}$).

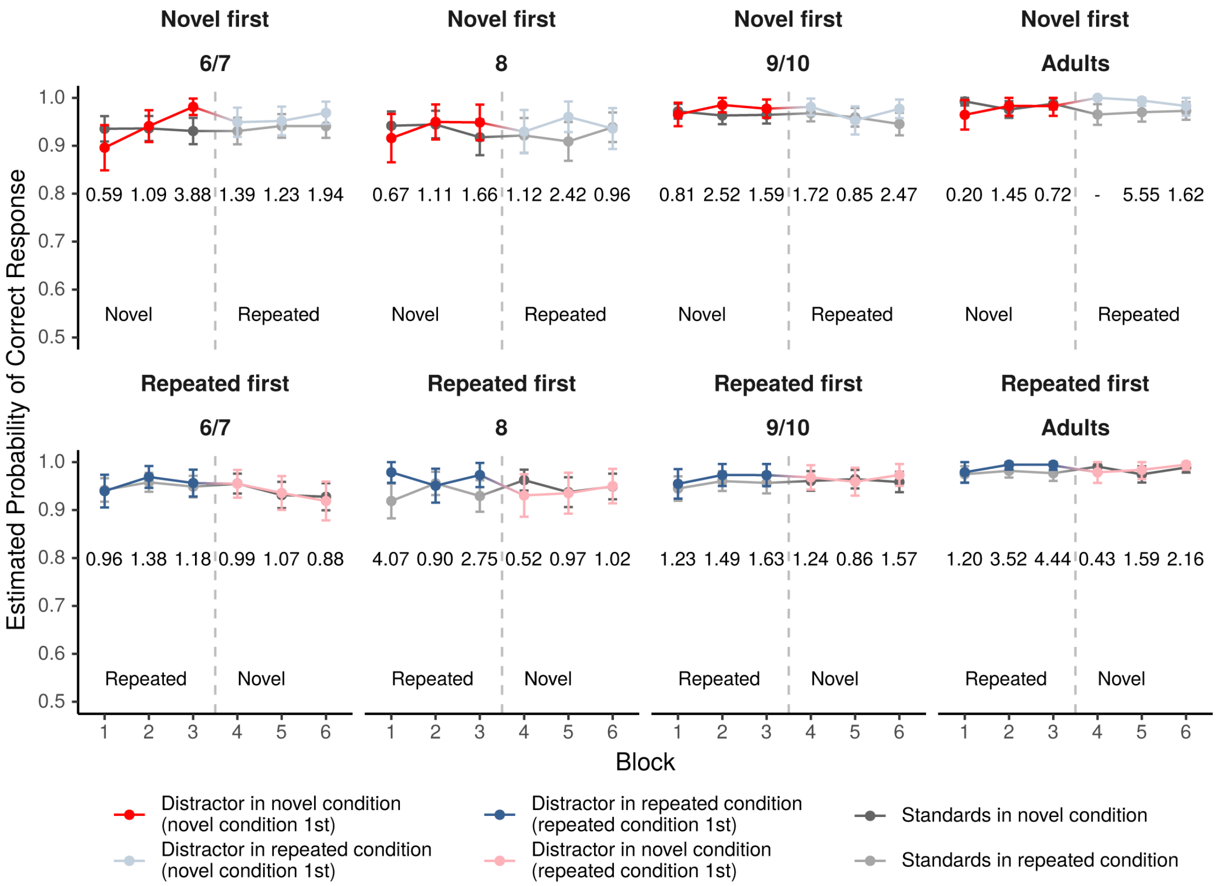

Figure A2.
Model-Implied Hit Rate of the Block-Effect Model as a Function of Experimental Block (1 to 6), Randomization (novel first vs. repeated first), Age, and Sound Type. Error bars represent 95% confidence intervals. The numbers at the bottom are odds ratios for distraction effects (i.e. $\frac{Odds_{distractor}}{Odds_{standard}}$).

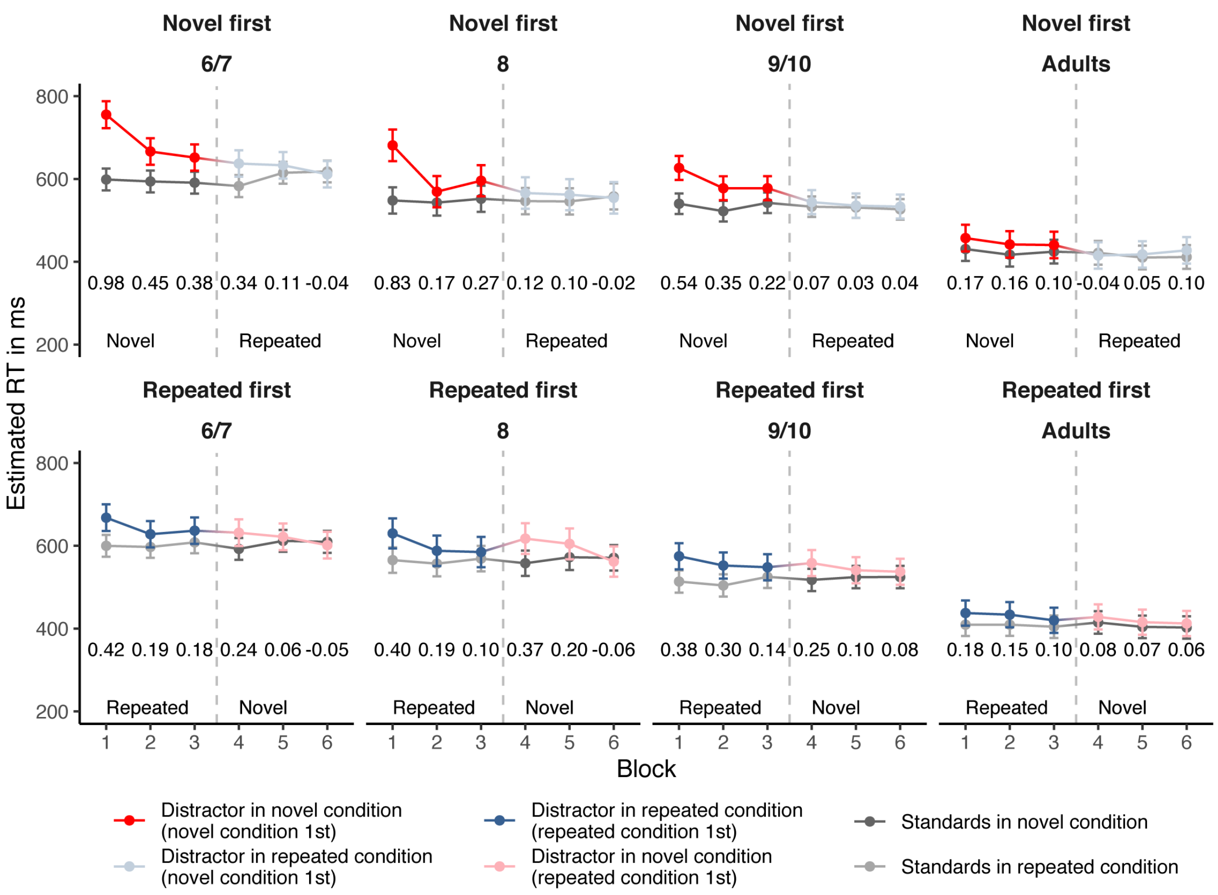

Figure A3.
Model-Implied RTs of the block effect Model as a Function of Block (1 to 6), Randomization (novel first vs. repeated first), Age, and Sound. Error bars represent 95% confidence intervals. The numbers at the bottom are standardized distraction effects (i.e. $\widehat{RT}_{distractor} - \widehat{RT}_{standard}$ for z-standardized RTs).

Part B

During the first recording sessions the repeated deviant sound was accidentally presented as novel in the novel condition. This has no implication when the participant starts with the novel condition as this sound was always new when firstly presented. In the following repeated condition, the distractor was never new as it was always presented several times in the training block. When the participant starts with the repeated condition then in the novel condition 1 out of 24 distractor sounds was not new. We therefore removed this single trial in the respective participants from further analyses. An age-matched comparison between samples with and without correction revealed no differences.

Part C

Sample size considerations in the context of linear mixed models are relatively complex because the sample size has to be considered on both levels of the analysis and many diverging recommendations were proposed [e.g., [1,2,3]]. Although power computations are possible via simulations for basically any mixed model [e.g., [4,5]], they require a priori knowledge regarding the many parameters of a mixed model (i.e., random effect (co-)variances and effect sizes of the fixed effects) that was not available for our study. We aimed at testing as many participants as possible but at least 30 participants per group. In the following, we present the results of some simulations that we conducted to investigate the sensitivity of our approach to the effects of interest.

In absence of specific a-priori knowledge, we investigated the sensitivity of our research design toward the hypothesized effects using power curve analyses. That is, we approximated the smallest effect size for which we would have reasonable power (i.e., 90%). In order to run a power simulation in a feasible amount of time using the *simr* package [[5]], we made the following simplifications to the model:

1) The residual correlations between adjacent trials were fixed to zero (as these were very small, this should not bias the results severely).
2) We estimated a homoscedastic model because the package *simr* does only include lme4 models for which this feature is not yet available.

We identified the following parameters as most central to our substantive hypotheses:

(1) for the condition effect model:
    a. pairwise comparison between distractors and standards within a specific group and condition
    b. pairwise comparison of distraction effects (i.e. RT(distractor) – RT(standard)) between novel and repeated condition within a specific group

c. pairwise comparison of distraction effects between two age groups within a specific condition

(2) for the block effect model:

a. pairwise comparison of distraction effects (i.e. RT(distractor) – RT(standard)) between two different blocks within a specific group

b. pairwise comparison of distraction effects within a specific block between two groups

For each of these parameters of interest, we approximated statistical power by simulating 1000 random samples and counting the number of significant effects over a range of effect sizes for the parameter of interest. In order to make the simulation as realistic as possible, the estimated model parameters were used as population parameters and only the parameter of interest was varied.

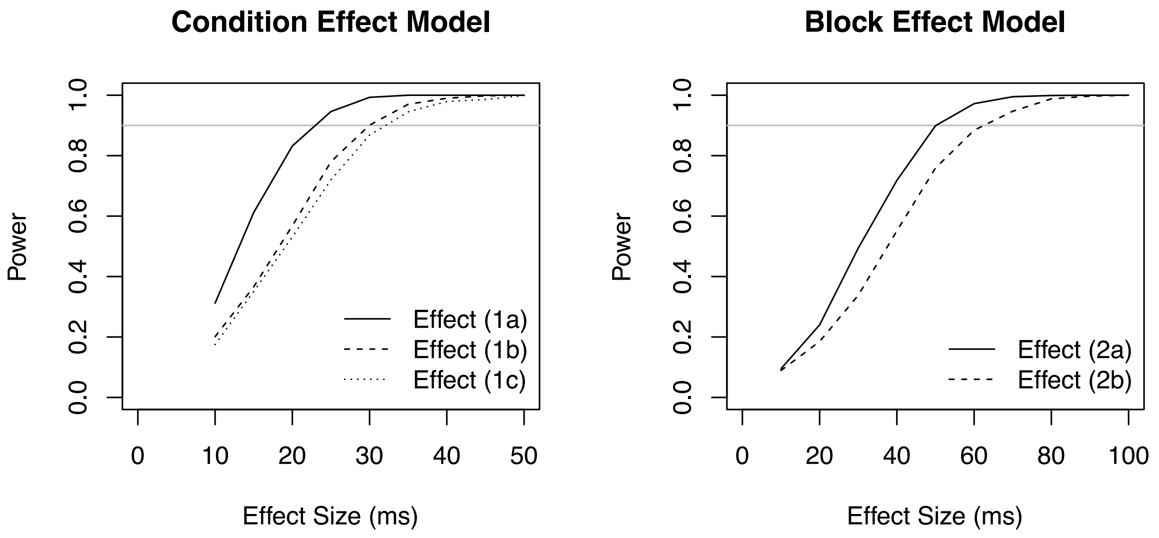

Figure C1. Results of the power simulation for the condition effect model (left panel) and the block effect model (right panel).

With respect to the condition effect model, the results demonstrated that our research design had sufficient power (i.e., 0.9) for the tested sample size for relatively small distraction effects

of 20 ms [Effect (1a)]. Regarding differences between conditions [Effect (1b)] and groups, [Effect (1c)] respectively, sufficient power was achieved for differences in distraction effects around 30 ms. For the more complex block effect model, power was lower, requiring differences in distraction effects between blocks [Effect (2a)] of about 40 ms within groups and about 60 ms between groups [Effect (2b)] for sufficient power.

Conclusion. The simulation demonstrates that our research design was especially suited to detect differences between the adults and the groups of children for which the effect sizes were well above the minimum effect size with sufficient power. Comparisons between the children groups, however, should be interpreted bearing in mind that the power was lower, implying that more detailed studies of developmental trajectories across childhood probably require samples even larger than the one tested here.

Regarding the block-wise analyses, the simulation implied that the effect sizes for the most important results (larger distraction effects in children in first block compared to adults) were by far larger than the minimum effect size for sufficient power. Conclusions regarding changes in distraction effects between further blocks (where differences were considerably smaller) and, again, regarding comparisons of children groups should made with caution. That is, the absence of significant differences in distraction effects between children groups should not necessarily be interpreted as a stagnating development.

Part D

An anonymous reviewer encouraged us to test an alternative interpretation for our finding of larger distraction effects in children than in adults. Specifically, it was suggested that the results pattern could have arisen simply from a general development towards faster processing. According to this reasoning, the reduced distraction effects could be the result of a simpler developmental process: With increasing age, processing becomes faster as indicated by reduced overall RTs. Hence, the absolute difference between distractor and standard sound RTs

decreases but *relative* to the standard RT, the distraction effect remains the same. Formally, such a *proportional* linear model implies equal distraction effects relative to the intercepts (i.e., the average standard RT) across all groups. Such a model can be construed as a restricted version of our full model with interactions in which an equality constraint is placed on the $\frac{\text{distraction effect}}{\text{average novel RT}}$ ratios across all groups. Because this restricted proportional model is nested within the full interaction model, it is possible to compare both models with a likelihood ratio test – investigating the hypothesis if a free interaction model fits the data substantially better than the proportional model. We conducted such an analysis using the software *Mplus* [Version 8, [6]]. We note that due to software limitations, it was not possible to model heteroscedasticity at the same time – however, the results of the free interaction model replicated the results of the heteroscedasticity-adjusted model closely including all parameter tests, thus we consider the test informative despite the neglected heteroscedasticity. Since our main analyses already established that children and adults differed from another mainly in the novel condition and that the distraction effects changed over the course of the experiment, the model comparison was conducted for the novel condition only, and both across all blocks and only across trials from the first block. The test indicated superiority of the free interaction model both across all blocks, $\chi^2(3) = 15.19$, $p = .002$, and for the first block only, $\chi^2(3) = 24.35$, $p < .001$. That is, a model in which distraction effects could vary freely between groups explained the data substantially better than a model in which the distraction effect was restricted to an equivalent $\frac{\text{distraction effect}}{\text{average novel RT}}$ ratios across all groups.

In a next step, we investigated the pattern of RT ratios further. Descriptively, the RT ratio was similar across children groups (all blocks / 1st block only; 6-7 years: 0.09 / 0.19, 8 years: 0.08 / 0.26, 9-10 years: 0.08 / 0.192) but different between children and adults (0.04 / 0.09). That is, the size of the distraction effects in children was about 9%/20% (all blocks / 1st block only) of a standard RT while it was only 4%/9%) in adults. This indicates that the larger distraction

effects in children indeed exceeded what would be expected based on a proportional model. Pairwise comparisons confirmed significant ratio differences (i.e., changes exceeding a proportional model) between children and adults (see Table D1). Overproportional differences between children groups could not be established. In light of power considerations (cf. Suppl. part C), this result is not surprising since smaller differences would be expected between children groups than between children and adults. We conclude that a proportional linear model is not sufficient to explain differences between children and adults.

Table D1

Pairwise comparisons of the distraction effect to novel RT ratios

	**all blocks**			**1st block only**		
Groups	ΔRatio	*SE*	*p*	ΔRatio	*SE*	*p*
6/7 vs. 8	0.011	0.022	0.317	-0.072	0.064	0.317
6/7 vs. 9/10	0.012	0.019	0.991	-0.001	0.050	0.991
6/7 vs. adults	0.052	0.017	**0.034**	0.100	0.042	**0.034**
8 vs. 9/10	0.000	0.021	0.281	0.071	0.054	0.281
8 vs. adults	0.041	0.019	**0.003**	0.172	0.050	**0.003**
9/10 vs. adults	0.040	0.015	**0.003**	0.101	0.030	**0.003**

Note. The *p*-values were FDR-corrected.

References

1 Angrist, J. D. & Pischke, J.-S. Mostly Harmless Econometrics: An Empiricist's Companion. (2009).

2 Gelman, A. & Hill, J. *Data Analysis Using Regression and Multilevel/Hierarchical Models*. (Cambridge University Press, 2007).

3 Snijders, T. A. B. & Bosker, R. J. *Multilevel Analysis: An Introduction to Basic and Advanced Multilevel Modeling*. (Sage Publishers, 2012).

4 Brysbaert, M. & Stevens, M. Power Analysis and Effect Size in Mixed Effects Models: A Tutorial. *Journal of Cognition* **9**, 1-20, doi: https://doi.org/10.5334/joc.10 (2018).

5 Green, P. & MacLeod, C. J. SIMR: an R package for power analysis of generalized linear mixed models by simulation. *Methods in Ecology and Evolution* **7**, 493-498, doi:10.1111/2041-210X.12504 (2015).

6 Muthen, L. K. & Muthén, B. O. *Mplus user's guide (7th ed.)*. (Muthén & Muthén, 2015).
